# A sports and health application for patients with type 1 diabetes mellitus -An end-user survey on expectations and requirements

**DOI:** 10.1007/s40200-022-01024-0

**Published:** 2022-05-03

**Authors:** Roman Holzer, Fabian Werner, Meinolf Behrens, Carsten Volkery, Christian Brinkmann

**Affiliations:** 1 Department of Preventive and Rehabilitative Sport Medicine, Institute of Cardiovascular Research and Sport Medicine, German Sport University Cologne, 50933 Cologne, Germany; 2 Diabeteszentrum Minden, 32427 Minden, Germany; 3 IST University of Applied Sciences, 40233 Düsseldorf, Germany

**Keywords:** MHealth, Type 1 diabetes mellitus, T1DM, Mobile application, Sports app, Health app

## Abstract

**Purpose:**

The aim of this survey is to investigate T1DM patients’ expectations for and requirements of an ideal mobile self-management app with a special focus on functions for sports and exercise.

**Methods:**

A total of 251 persons participated in the survey. After checking for completeness and plausibility, the answers of 167 patients diagnosed with T1DM (66% female, 34% male) were analyzed.

**Results:**

The key features/aspects that were identified as being “rather important”/”very important” by more than 75% of respondents are: data security (96.4%), integration of further health data (e.g., heart rate, step count, calories) from other apps already installed on their smartphone (92.2%), automatic import of glucose data from other apps (91.6%), individual target setting (87.4%), warnings about abnormal glucose levels (82.6%), warnings about other abnormal health data (81.4%), diary function (80.8%), information on the training session after the workout (80.8%) and displaying/processing of further fitness variables (such as heart rate, step count, etc.) from other health-related wearable systems (77.8%).

**Conclusions:**

This study identifies the most relevant features of an ideal self-management app with functions for sports and exercise targeted at patients with T1DM that should be considered in the development of such an app.

## Introduction

The progress of digitalization has a significant impact on the management of diabetes mellitus. The American Diabetes Association (ADA) divides diabetes technology into two basic categories: for administration of insulin, e.g., via insulin pens or pumps, and for the analysis of glucose concentrations, e.g., via glucose meters or continuous glucose monitors (CGM) [1]. Diabetes technology, however, also includes closed-loop systems, telemedicine, electronic medical records and mobile health (mHealth) applications (apps) [2].

Many glucose measurement and insulin administration systems can be controlled via mobile apps, and even the range of functions of initially analog devices, such as insulin pens, is widening through digitalization and interconnectivity [2]. In fact, many diabetes technology manufacturers have developed own mobile applications that allow their devices to connect to smartphones and help customers record, manage and analyze their data.

However, independent of diabetes management devices, there are numerous (commercially) available mHealth apps for diabetes mellitus patients [3–5]. mHealth apps can generally be divided into three categories: apps (1) that are used to monitor well-being; (2) that function as stand-alone medical devices, and (3) that display, download and/or use data from medical devices to diagnose, prevent, monitor or treat a condition [3].

Ideally, diabetes mobile apps support users in managing their disease and in preventing diabetes-related complications, thereby improving their overall quality of life. Numerous meta-analyses and systematic reviews have shown potential benefits of mHealth apps on glycemic control in patients with diabetes mellitus, resulting in a reduction of glycated hemoglobin (HbA1c) levels [2, 6–11]. It is difficult to determine the actual effectiveness of the use of mHealth apps due to the combinations of different features and the fact that many studies are time limited and have small samples [2, 3]. Long-term outcomes, in particular, have not yet been sufficiently investigated. The currently available results, however, point to the high potential and benefits of digital diabetes technology.

Regular physical activity is an elementary part of a healthy lifestyle for both T1DM and T2DM patients [12]. Regular exercise improves, among other variables, cardiorespiratory fitness, insulin sensitivity, glycemic control, body composition, lipid profiles, blood pressure and overall mortality risk [12]. Exercise poses an enormous challenge for T1DM patients due to their individual insulin therapies and varying physiological responses to different exercise types [13]. Avoiding severe hypo- and/or hyperglycemic conditions during exercise is thus a complex undertaking for individuals with T1DM and mHealth applications could provide helpful assistance in this regard [5, 14].

Managing diabetes is a complex, lifelong task that impacts all areas of life. Among others, mHealth apps for diabetes include the following management tasks: glucose monitoring, insulin delivery, nutrition, physical activity, facilitation of communication with healthcare providers or diabetes support groups, and education [2–4, 15].

Available apps usually only cover between one and three of the above mentioned diabetes management tools, but not the entire range [3, 15]. Patients with diabetes must therefore rely on more than one app to cover all relevant aspects of diabetes management. This study aims to identify important variables of an optimal stand-alone mobile app for the self-management of diabetes with a special focus on sports and exercise. The results are expected to provide practical guidance for the development of future mHealth applications for patients with T1DM.

## Materials and Methods

The quantitative survey was conducted among T1DM patients. An online version of the questionnaire was made available on “surveymonkey.com”. It took approximately 10 min to complete the questionnaire. Data were collected in Germany over a 3-month period from October to December 2019.

### Subjects

For inclusion in the survey, participants had to be diagnosed with T1DM. No further exclusion criteria were defined to elicit the highest possible number of responses. Recruitment took place via leaflets in outpatient diabetes treatment and education centers and social media. All subjects participated voluntarily without monetary incentive and provided informed consent for inclusion in the study.

### Conceptualization of the Questionnaire

To conceptualize the questionnaire, existing diabetes apps were analyzed in terms of functions and content. The top three rated apps available from the Apple App Store were used. We reviewed the functions each individual app includes to facilitate day-to-day diabetes management and what types of diabetes-specific content (information, guides, etc.) are available. The final questionnaire was constructed based on these findings. For the assessment of statements, 4-point Likert rating scales were used [16]. The Introduction informed the participants about the purpose of the study, the approximate time required to complete the survey, confidentiality, data privacy and researchers’ contact details. 

### Data Analyses

Before conducting the statistical analyses, the participants’ responses were checked for plausibility. Implausible or inconsistent answers were not considered in further analyses. The collected data were analyzed descriptively. Values are expressed as mean values ± standard deviation or as mean values and 95% confidence interval (95%-CI). In the evaluation, functions which were rated as “rather important” or “very important” by at least 75% of the participants were deemed particularly relevant. The graphs were prepared using Microsoft Office Excel^®^ 2016 for Windows (Microsoft Corporation, Washington, USA).

### Ethical Approval

The study was in accordance with the regulations of the Ethics Committee of the IST University of Applied Sciences and the guidelines of the Declaration of Helsinki. Patients gave their informed consent by clicking on the webpage. The patient data and responses were recorded anonymously.

## Results

### Anthropometric and Sociodemographic Data

A total of 251 subjects participated in the survey. After verifying the inclusion criteria and completing a plausibility check, 167 respondents were included in the analyses. Reasons for exclusion were either missing T1DM as well as incomplete or inconsistent responses. Among the included subjects, 65.9% were female and 34.1% were male. The mean age of the sample was 33 ± 12 years (min-max: 8–65 years). The majority of respondents were employees (61.7%), and 6.0% were self-employed. Among the participants, 16.2% were university students at the time of the survey. One respondent (0.6%) was retired. The remaining participants (15.6%) had other employment, such as school pupil or homemaker. The monthly net income of 58.1% of participants was between EUR 1,000 and EUR 3,000. 18.6% reported a lower and 10.2% a higher monthly net income. 13.2% did not provide any information.

In terms of physical activity, most participants described themselves as casual exercisers with 1–2 workouts per week (43.1%) or recreational exercisers with 3–4 workouts per week (40.1%). 4.2% of the participants described themselves as competitive athletes (daily training), 12.6% were non-active persons. 41.9% spent 1–3 h per week engaging in physical activity, 28.1% 3–5 h and 19.2% even more than 5 h per week. Only 10.8% of participants engaged in physical activity of less than 1 h per week. 7.2% worked in the field of sports as trainers /coaches.

A total of 79.0% of participants said their diet was extremely healthy (3.0%) or somewhat healthy (76.0%), while 21.0% stated that their diet was somewhat unhealthy. No one rated his/her diet as unhealthy. The majority of participants were non-smokers (80.8%), while 19.2% smoked regularly (10.8%) or at least occasionally (8.4%).

64.7% of participants used CGM systems exclusively to monitor and control their glucose concentrations, while only 9.6% used glucose meters exclusively. All other participants used a mix of different glucose measurement options.

### General Aspects

Most participants were users of either Apple iOS (41.3%) or Google Android (58.1%). The number of smartphone apps used by the respondents ranged from 1 to 10 (25.1%), 11–20 (30.5%), 21–30 (26.3%), 31–40 (12.0%), or was over 40 (6.0%).

Most of them (83.8%; 95% CI: 78.2 − 89.4%) strongly agreed or rather agreed with the statement that there is a general need for a sports health app specifically for T1DM patients. 73.1% (95% CI: 66.4 – 79.8%) affirmed that they had a personal need for such an application. The fields of application (multiple answers possible) ranged from “use in sports” (78.4%; 95% CI: 72.2 – 84.7%), “use at work” (25.1%; 95% CI: 18.6 – 31.7%) or “use in private daily life” (67.7%; 95% CI: 60.6 – 74.8%), and “use on vacation” (28.1%; 95% CI: 21.3 – 35.0%). In this regard, 48.5% (95% CI: 40.9 – 56.1%) would use the application at least once a day. 37.7% (95% CI: 30.3 – 45.1%) would use it several times a week, while 13.8% (95% CI: 8.5 – 19.0%) would use it less than 3 times a week.

The maximum price the majority of respondents would pay for such a sports health application ranged from EUR 1–10 (1–5 EUR (35.3%; 95% CI: 28.1 – 42.6%), EUR 5–10 (35.3%; 95% CI: 28.1 – 42.6%)). 8.4% (95% CI: 4.2 – 12.6%) would want to spend less, and 9.6% (95% CI: 5.1 – 14.0%) would spend more money on it. The remaining 11.4% (95% CI: 6.6 − 16.2%) would not purchase such an app at all.

### Functionality of the Sports and Health App

**Fig. 1:**
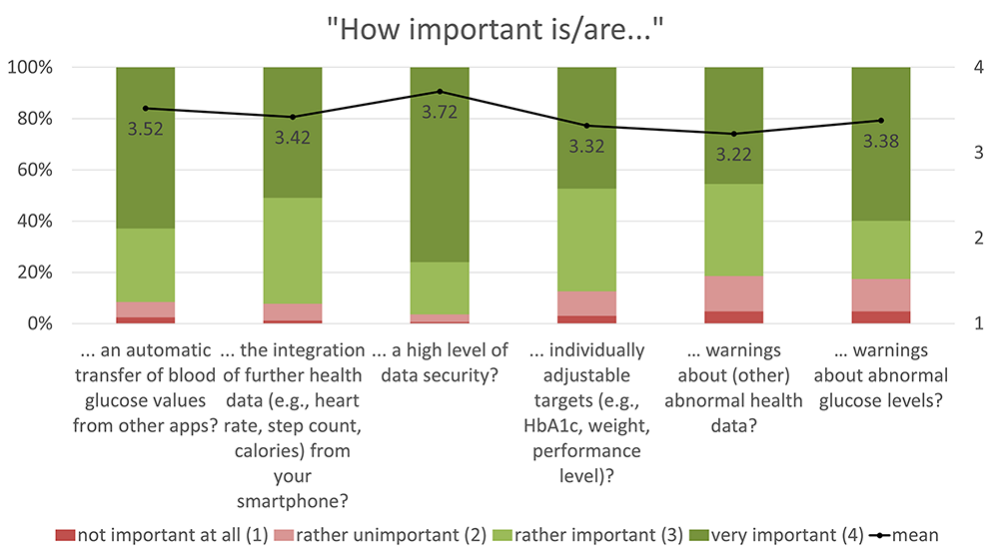
Ratings of **technical features/aspects**. Mean values are shown in the bars

**Fig. 2:**
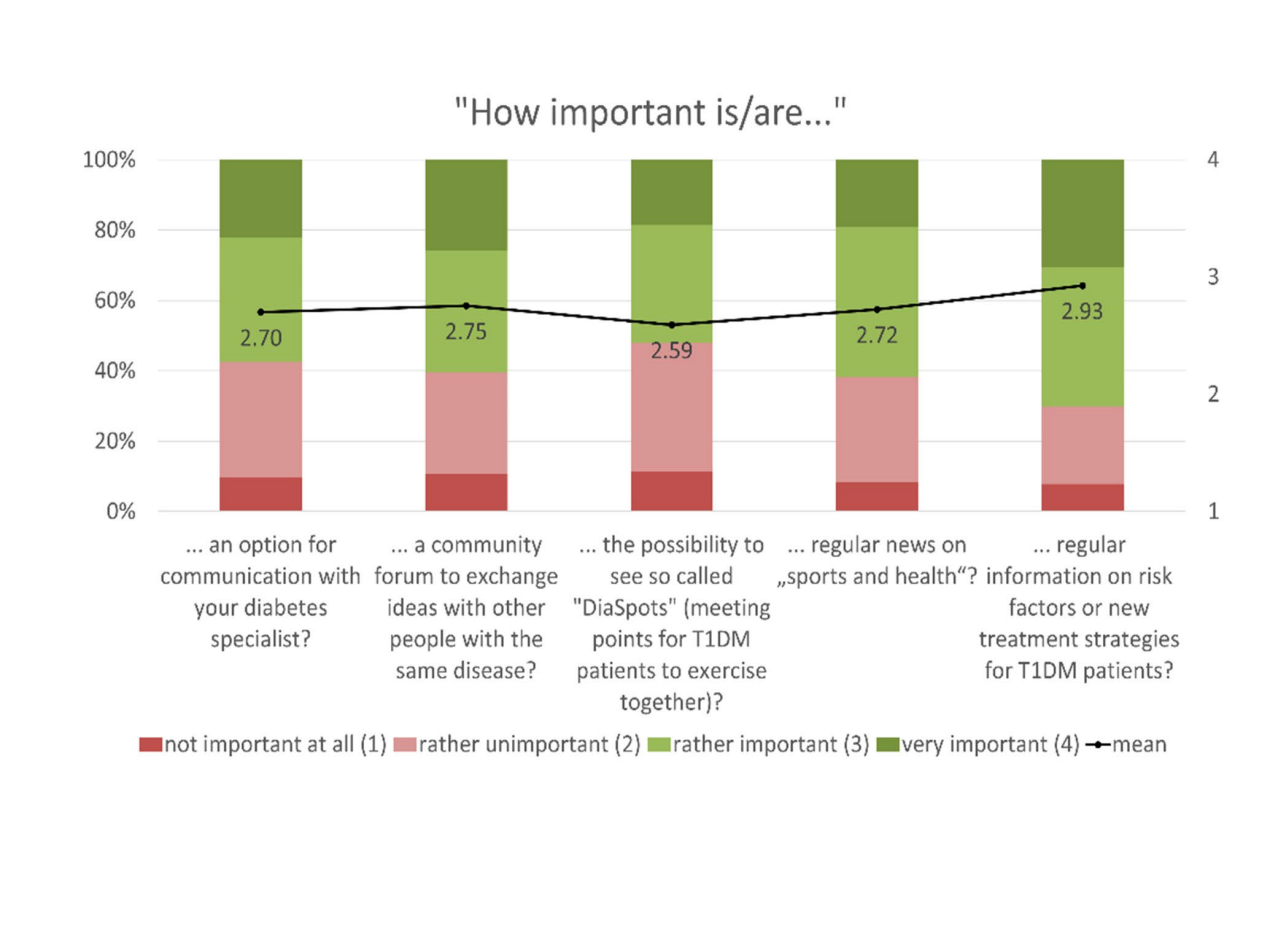
Ratings of **communication and information features/aspects**. Mean values are shown in the bars

**Fig. 3:**
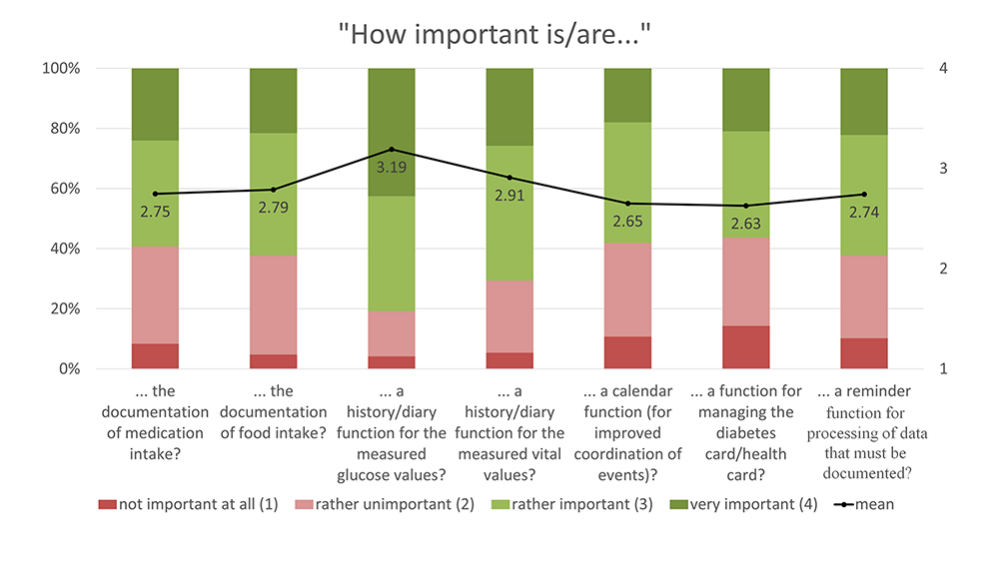
Ratings of **documentation features/aspects**. Mean values are shown in the bars

**Fig. 4:**
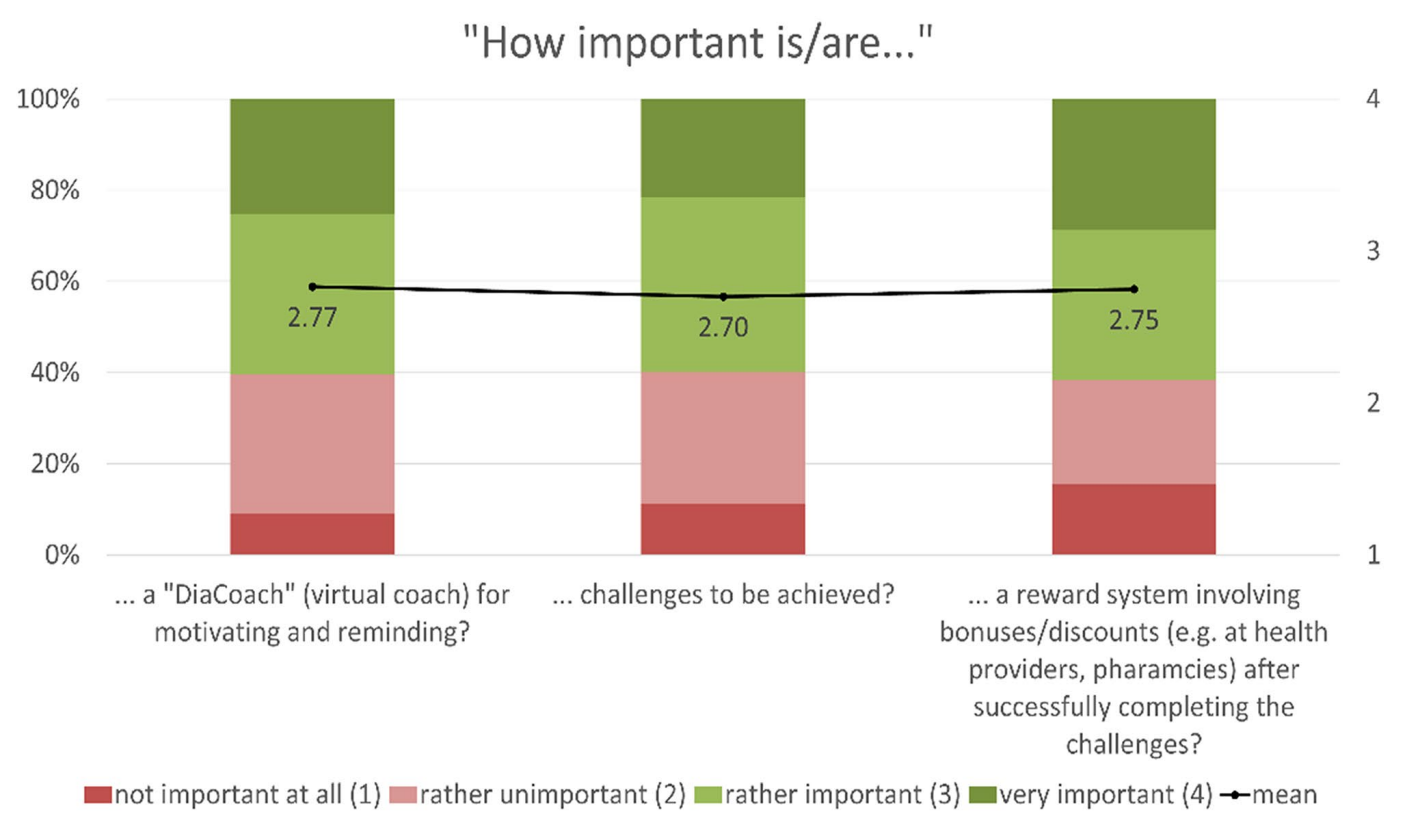
Ratings of **motivational features/aspects**. Mean values are shown in the bars

**Fig. 5:**
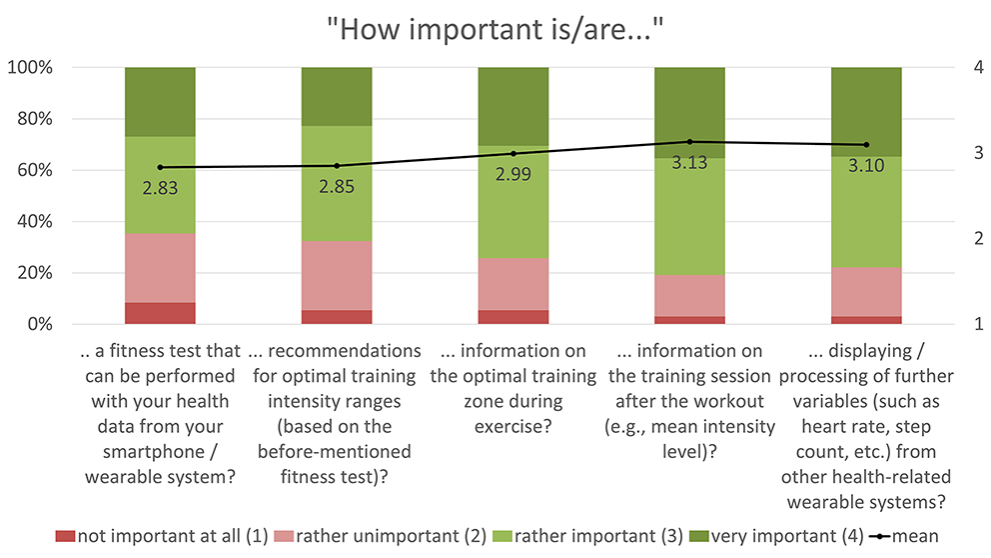
Ratings of **support features/aspects**. Mean values are shown in the bars

The expectations of and requirements for such a sports health app were mainly determined using 4-point Likert rating scales: (1) not at all important, (2) rather unimportant, (3) rather important, (4) very important. Based on the numerical rating, the mean values of the individual statements were calculated and are presented together with the proportional distribution in Figs. 1, 2, 3, 4 and 5. The respondents’ expectations and requirements were divided into different categories: technical aspects, communication and information, data documentation, motivation and exercise support.

### Key Features of the Sports and Health App

The key features/aspects that were deemed “rather important”/”very important” by over 75% of respondents were: data security (96.4%), integration of further health data (e.g. heart rate, step count, calories) from other apps on their smartphone (92.2%), automatic import of glucose data from other apps (91.6%), individual target setting (87.4%), warnings about abnormal glucose levels (82.6%), warnings about other abnormal health data (81.4%), diary function (80.8%), information on the completed training session (80.8%) and displaying/processing of further fitness variables (such as heart rate, step count, etc.) from other health-related wearable systems (77.8%).

## Discussion

The survey results provide useful information on desirable features of a T1DM self-management app with functions for sports and exercise.

The survey identified a glucose diary as one of the desirable key functions of a self-management app for diabetes mellitus, which is in line with another survey on diabetes self-management apps [17]. In that survey, the glucose diary was found to be one of the most desirable functions. Interoperability was also deemed particularly important by respondents in terms of the integration of glucose data which should preferably be automatically transferred from other apps or glucose sensors to the self-management app, thus reducing manual entries. These functions are currently only being offered by a limited number of available diabetes apps [18]. Interoperability and integration of different functions and features could therefore be a decisive factor for the success of future mHealth apps. The trend toward more integration and increased automation already appears to be gaining momentum [3] and, according to the results of this survey, is also sought by T1DM patients.

Individual target setting (e.g., HbA1c, weight, exercise duration, etc.) and app support in assessing health data seem to be of particular interest for the participants. It is important for many participants to be able to individually set reminders, notifications and warnings. These functions have also been identified as key features in another study and in addition to reminders for glucose measurement or insulin delivery or doctor’s visits [18].

The respondents first and foremost expect a comprehensive sports and health app for T1DM patients to integrate relevant health and fitness variables (such as step count, heart rate, etc.) and to provide individualized and up-to-date support to manage their disease. Although the education and support feature was also rated as an important feature by the participants in this study as well as in a comparable study [19], only few apps take this aspect into account.

Decision support on the current evidence base is one of the major problems of many diabetes apps. Diabetes mobile apps are mostly unregulated [2–4, 20–22] and unfortunately, many do not comply with evidence-based guidelines or have simply become outdated due to a lack of updates [3, 15, 20]. This might pose a high health risk for users, as miscalculations or misinterpretations can have serious consequences.

The lack of regulation also has an impact on data security. A high level of data security is a key factor in a digital world [3] and was also the most highly rated item in this survey. Accordingly, there is a high demand among participants for protection of personal health-related data. Indeed, many mHealth applications fail in this regard. Lack of encryption, insecure data transmission or inadequate programing practices are some of the major security concerns [23, 24]. At the same time, many diabetes apps share data with third parties without notifying users [25]. However, the problem of lack of data security is not limited to the disclosure of sensitive personal health data; a manipulation of data can pose a health risk if it results in the administration of incorrect insulin doses, for example. Consequently, data security is a crucial factor in the development of a diabetes app.

Many T1DM patients seek support for sports and physical exercise. In healthy individuals, smartphone-based physical activity interventions appear to have positive effects on improving physical and psychosocial factors [26]. For individuals with T1DM, however, physical activity is a complex undertaking in terms of maintaining glucose homeostasis. The results of this survey demonstrate the need for support during physical activities. In this regard, both active support during exercise/sports and for the subsequent interpretation of data are important factors for participants. Information could assist T1DM patients’ decisions in terms of glucose regulation (such as glucose intake or insulin dosing). To date, only a few apps provide active support for T1DM patients during physical activity.

The respondents’ ratings of motivational features such as a virtual (diabetes) coach, challenges or a reward system were ambivalent. Some of the respondents considered them to be quite inspiring or could at least imagine that such features might motivate users to pursue a healthy lifestyle, even if they themselves would not use them. Others, however, saw no need for the inclusion of motivational features or expressed concerns about social pressure, frustration and data privacy.

The relatively narrow confidence intervals calculated in this study show a comparatively low degree of uncertainty for estimates in the true population.

Some limitations must be considered. One of the limitations of this study is the wide age range of the participants, which at the same time covers a large group of potential users. It is irrefutable, however, that 8-year old patients cannot be compared to 65-year-old patients in terms of application use.

Although numerous factors were considered that may have influenced response behavior, some may be missing (e.g., mobility of the participants).

Future research should focus on how the issues described can be addressed and implemented in the development of mHealth applications. Furthermore, once such a self-management app for T1DM patients has been developed, its effectiveness should be evaluated in clinical trials, which unfortunately has rarely been the case with the currently available mHealth apps for diabetes management [27].

## Conclusions

The results of this survey study illustrate that T1DM patients seek a diabetes health app that integrates many relevant factors and functions, such as glucose monitoring, insulin delivery, nutrition, physical activity, etc. in a single app and that allows for interoperability with other apps. Support during sports/ physical exercise is a key feature that should be considered in the development of an ideal app. A high level of data security must be guaranteed to prevent misuse of sensitive personal data.
